# Editorial

**DOI:** 10.1002/osp4.19

**Published:** 2015-12-23

**Authors:** David B. Sarwer

**Affiliations:** ^1^ Obesity Science and Practice

I am eager to write to you, as the fall of 2015 has been an exciting time for *Obesity Science & Practice*. The first issue of the journal was produced and made available earlier this fall. We published seven articles, and I am thrilled with their breadth of scope and high quality. The papers reported on studies in both paediatric and adult obesity as well as studies of eating behaviour and physical activity. Our first publications include results from large randomized controlled trials and high quality observational studies. Two papers reported on findings from seminal studies in the field, including LookAhead and the POWER trials. Many of them were authored by investigators at some of the worlds most well‐known and respected obesity research programs. I believe that these first papers have set the tone for the type and quality of papers we look to publish.

Within the past year, 57 manuscripts were submitted to *Obesity Science & Practice*. Seventeen were directly submitted to the journal. The other 40 were referred in from the other obesity journals published by Wiley: *Obesity*, *Obesity Reviews*, *Pediatric Obesity* and *Clinical Obesity*. Authors submitting to *Diabetes Obesity and Metabolism* now have the opportunity to transfer their papers to *Obesity Science & Practice* as well. Many of these papers underwent peer review at these publications and, for a number of reasons, just missed the cut for publication. The editors of those journals, Drs Eric Ravussin, David York, Michal Goran and Nicholas Finer, in their communications with the authors, indicate that direct referral to *Obesity Science & Practice* is an option. When papers come to us with reviews from those journals, I conduct a preliminary review of the paper. Based on that review, I make a determination on whether to have the authors revise the manuscript based on the previous reviews or I send the paper out to our editorial board for further review. The first option, which I prefer, streamlines the review process for authors and, over time, will likely decrease the time to publication for those papers. I want to thank my fellow Wiley Editors‐in‐Chief for their active participation in this process, which has helped us to our strong start.

Many of you have likely just returned from Obesity Week in Los Angeles. I was impressed by the high quality oral presentations in both the American Society for Metabolic and Bariatric Surgery and The Obesity Society sessions, as well as the number of strong posters found in the joint poster sessions. Many of you who authored work at the meeting now are writing your manuscripts. I invite you to consider *Obesity Science & Practice* as an outlet for your scholarship. Given our relationships with the societies that hosted Obesity Week, as well as World Obesity, your work will be read by an international, multidisciplinary audience of your peers. Wiley emphasizes quality as do I, so I am confident that your work will be published in good company.

Speaking of Wiley, I want to take a moment to thank the Wiley/*Obesity Science & Practice* team for their hard work this past year. This includes Sarah Gelotte, who works closely with me on the day‐to‐day operations of the journal, Gio Monteiro, Helen Eassom and Jonathan Rappay. I also want to welcome Joanna Ventikos, our journal publishing manager, and Emily Borromeo, our new editorial assistant to the team. Gio, Joanna and I had a chance to meet in person in Los Angeles. In addition to reviewing the progress of the journal, we also talked about our shared interest in James Bond movies and the recently released Spectre. I came away from that meeting knowing that we have a great team in place that will further ensure the success of the journal.

I mentioned that this has been an exciting fall for the journal. It has also been an exciting fall for me professionally. After 20 years at the Center for Weight and Eating Disorders at the University of Pennsylvania, I accepted the position of Associate Dean for Research and Director of the Center for Obesity Research and Education at the College of Public Health at Temple University. I am excited to take on the challenges of these new positions and thrilled that I am able to do so without having to relocate from the Philadelphia area, which has been home for me and my family since 1995 (although as many of you know, I am still a Chicagoan at heart). I want to thank my colleagues at Penn for their years of support and friendship, which I am sure will not change with the move just 5 miles away. I also want to thank my new colleagues at Temple for the warm welcome they have given me. I am eager to work with them to further develop and grow an already well‐recognized team of high quality researchers.

While I am busy learning my new responsibilities, I anticipate no changes in the operations of *Obesity Science & Practice.* I hope to be able to release our second issue later in 2015 or early in 2016. I look forward to seeing some of your work come across my desk in the near future.

Let me also be one of the first to wish you and yours the happiest of holidays and all the best for the new year.

